# Heterotopic autotransplantation of ovarian tissue in a large animal model: Effects of cooling and VEGF

**DOI:** 10.1371/journal.pone.0241442

**Published:** 2020-11-04

**Authors:** Samara S. Souza, Benner G. Alves, Kele A. Alves, Fabiana A. S. Brandão, Danielle C. C. Brito, Melba O. Gastal, Ana P. R. Rodrigues, José R. Figueireod, Dárcio I. A. Teixeira, Eduardo L. Gastal

**Affiliations:** 1 Laboratory of Diagnostic Imaging Applied to Animal Reproduction, Faculty of Veterinary Medicine, State University of Ceara, Fortaleza, Ceara, Brazil; 2 Laboratory of Manipulation of Oocytes and Preantral Follicles, Faculty of Veterinary Medicine, State University of Ceara, Fortaleza, Ceara, Brazil; 3 Department of Animal Science, Food and Nutrition, Southern Illinois University, Carbondale, Illinois, United States of America; University of Florida, UNITED STATES

## Abstract

Heterotopic and orthotopic ovarian tissue autotransplantation techniques, currently used in humans, will become promising alternative methods for fertility preservation in domestic and wild animals. Thus, this study describes for the first time the efficiency of a heterotopic ovarian tissue autotransplantation technique in a large livestock species (i.e., horses) after ovarian fragments were exposed or not to a cooling process (4°C/24 h) and/or VEGF before grafting. Ovarian fragments were collected *in vivo* via an ultrasound-guided biopsy pick-up method and surgically autografted in a subcutaneous site in both sides of the neck in each mare. The blood flow perfusion at the transplantation site was monitored at days 2, 4, 6, and 7 post-grafting using color-Doppler ultrasonography. Ovarian grafts were recovered 7 days post-transplantation and subjected to histological analyses. The exposure of the ovarian fragments to VEGF before grafting was not beneficial to the quality of the tissue; however, the cooling process of the fragments reduced the acute hyperemia post-grafting. Cooled grafts compared with non-cooled grafts contained similar values for normal and developing preantral follicles, vessel density, and stromal cell apoptosis; lower collagen type III fibers and follicular density; and higher stromal cell density, AgNOR, and collagen type I fibers. In conclusion, VEGF exposure before autotransplantation did not improve the quality of grafted tissues. However, cooling ovarian tissue for at least 24 h before grafting can be beneficial because satisfactory rates of follicle survival and development, stromal cell survival and proliferation, as well as vessel density, were obtained.

## Introduction

Ovarian tissue transplantation (OTT) has been used as a promising technique for the restoration of endocrine function and fertility in humans [1]. In this regard, OTT can be applied (1) for cancer patients prior to undergoing gonadotoxic therapy [2], (2) to animal models before clinical trials in human medicine [3, 4], and (3) as an alternative for preserving endangered animal breeds and species of high genetic value [5, 6].

The horse has been a suitable model for reproductive studies due to the striking similarities between women and mares related to follicular waves and hormonal changes [7, 8], anovulatory dysfunctions [9], and preantral follicle features [10, 11]. The use of heterotopic autotransplantation and xenotransplantation in livestock may become a powerful tool for (1) treatment of infertility caused by ovarian abnormalities (e.g., benign tumors and adhesions) and oviductal and uterine disorders, and (2) making easier the recovery of immature or mature oocytes from females of high genetic value that need preservation. In the area of preantral follicle research in horses, several studies have been conducted recently on ovarian tissue storage and cryopreservation [12–14], *in vitro* follicle culture [15, 16], and preantral follicle population, addressing aspects related to aging effect [17], follicular dynamics [11], and spatial follicle distribution [18].

Ischemia and reperfusion injuries induced by slow tissue revascularization shortly after grafting [19, 20] may lead to losses in primordial follicles [21] and stromal cells [22], along with tissue apoptosis and fibrosis [23]. Therefore, various strategies have been applied to accelerate graft revascularization and reduce hypoxia in autografted [24–26], allografted [27], and xenografted [3, 28–30] ovarian fragments.

Vascular endothelial growth factors (VEGF-A, VEGF-B, VEGF-C, VEGF-D, and placental growth factor, PIGF) are members of the superfamily of the platelet-derived growth factors, which are potent angiogenic factors that regulate neovascularization [31] and can be produced by the vast majority of cells under hypoxic condition or in tissues under growth and remodeling [32, 33]. The beneficial effects of VEGF on ovarian tissue transplantation have been demonstrated in sheep [34], cattle [4], dogs [35], humans [36, 37], and mice [24]. Furthermore, VEGF can accelerate angiogenesis and improve the ovarian cortex viability by limiting ischemia in xenografted sheep ovarian cortical pieces [3, 28].

Considering that ovarian sample collection is usually performed far from reproductive centers, a major obstacle for fertility preservation is the proper conservation and transport of the ovarian tissue. Several studies have been conducted regarding transportation or storage of fresh specimens to preserve and further optimize the use of the ovarian reserve [13, 38, 39]. In this context, induced hypothermia to approximately 4°C has been the most common approach used for transportation of ovaries to be preserved in horses [13], goats [40], cattle [41], and humans [42]. Therefore, we hypothesized that the addition of VEGF to a holding medium would improve ovarian tissue viability after storage at a cooling temperature (4°C) for 24 h before ovarian fragment autotransplantation. Thus, this study aimed to evaluate the efficiency of a heterotopic ovarian tissue autotransplantation technique in horses after ovarian fragments were submitted or not to cooling and/or exposed to VEGF.

## Materials and methods

### Chemicals

The chemical reagents used in this study were obtained from Sigma Chemical Co. (St. Louis, MO, USA) unless otherwise indicated.

### Animals

The experimental procedure was approved by the Ethics Committee for the Use of Animals of the State University of Ceará (research protocol #6520796/2015). Eight healthy crossbred cycling mares 7–22 years old and weighing between 350–400 kg, belonging to the Dr. Esau Accioly Vasconcelos Experimental Agricultural Farm of the Faculty of Veterinary, State University of Ceará, were used for the present study. Animals were kept on pasture, supplemented with balanced grain ration, minerals, and water *ad libitum* in a sheltered area. After the study, the animals were housed in the same facility and used for further research studies since the techniques used herein do not affect the reproductive activity of the animals.

### Ovarian tissue collection

**Fig 1:**
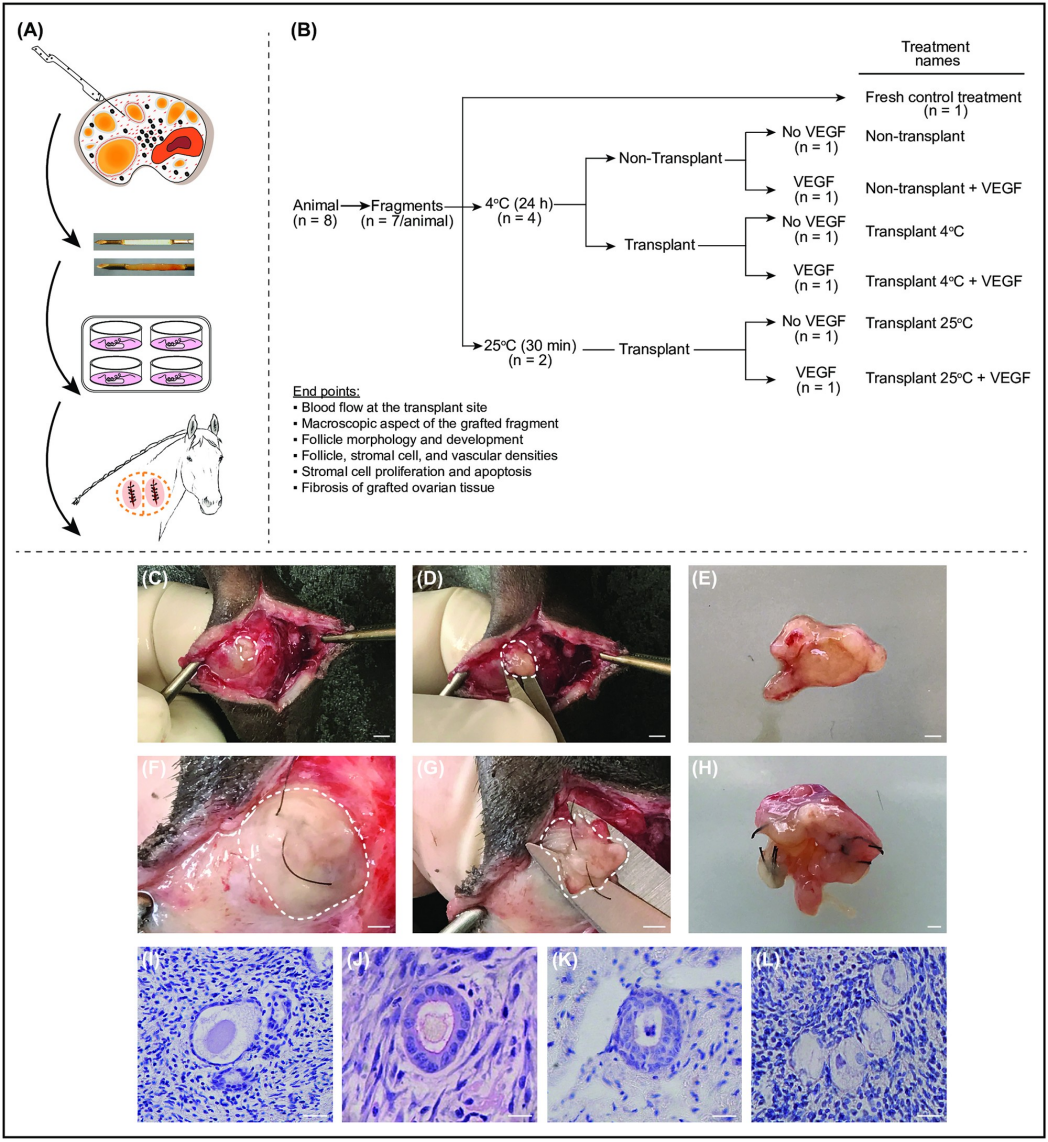
Illustration of experimental procedures performed to assess the effects of cooling and VEGF on heterotopic ovarian tissue autotransplantation in mares. (A) Collection of ovarian fragments by ultrasound-guided transvaginal biopsy pick-up method; biopsy needle notch without and with a harvested ovarian fragment; fragments with surgical thread ready to be transplanted; and two transplanted fragments. (B) Experimental design, treatments, and end points. (C–H) Representative images of ovarian grafts (white dashed circles) during the harvesting process at day 7. (C–E) and (F–H) Images obtained from two different animals. (E and H) Magnified images of recovered grafts. (F) Ovarian fragment embedded and covered with subcutaneous tissue. (C, D, G) Exposed grafts at the surgical site. (C, D, F, G) Bars = 3 mm, and (E, H) bars = 1 mm. (I–L) Representative images of morphologically (I–K) normal and (L) abnormal preantral follicles in grafted ovarian tissue previously cooled. (I, L) Primordial, (J) primary, and (K) secondary follicles. Magnification = 400×; bars = 20 μm.

Ovarian fragments were obtained via the biopsy pick-up (BPU) method (Fig 1A) [10, 15, 43]. Briefly, before each biopsy procedure, analgesia (flunixin meglumine; Banamine, 1.1 mg/kg, i.v.; Merck Animal Health, New Jersey, USA), rectal relaxation (hyoscine N-butyl bromide; Buscopan, 0.2 mg/kg, i.v.; Boehringer Ingelheim; Ingelheim am Rhein, Germany), and sedation (xylazine; Sedomin, 1 mg/kg, i.v.; König S.A, Argentina) were induced. The BPU device used was an automated spring-loaded instrument with an inner trocar point plunger containing a fragment notch (15 mm × 1.6 mm; Fig 1A) surrounded by an outer 16-gauge cutting needle (US Biopsy, Franklin, IN, USA). The device was introduced through a needle guide mounted on a probe handle with a 5 to 10 MHz transvaginal ultrasound-guided convex array transducer (Honda HCV-3710MV), which was used for placement of the biopsy needle within the ovary.

### Ovarian tissue handling

Immediately after the BPU procedure, one fragment from each animal was fixed for further histological analyses, while the remaining fragments were rinsed in MEM-HEPES (minimal essential medium) supplemented with penicillin (100 μg/ml) and streptomycin (100 μg/ml). Then, the fragments were transferred to 4-well culture dishes (Fig 1A) containing 1 ml of α-MEM supplemented with 1.25 mg/ml bovine serum albumin (BSA), 100 μg/ml penicillin, 100 μg/ml streptomycin, 0.047 mM sodium pyruvate, and 2.5 mM HEPES (holding medium). The fragments were then maintained in the holding medium without or with the addition of VEGF at 50 ng/ml (V7259; Sigma Aldrich, Saint Louis, USA) either at room temperature for 30 min or cooled at 4°C for 24 h [13, 29].

### Surgical procedure of grafting and recovery of ovarian fragments

Ovarian fragments were autografted in both sides of the neck in each animal (Fig 1A). Fresh and cooled fragments were grafted into the left (day 0) and right (day 1) sides of the neck, respectively. For the surgical procedure, the mares were sedated (xylazine; Sedomin, 1 mg/kg, i.v.; König S.A, Argentina) and anesthesia (lidocaine hydrochloride, 10 mg/kg, s.c.) was administered in the cervical-lateral portion of the neck. Then, a single incision (~4 cm) was made on the skin to engraft the ovarian pieces in a subcutaneous site. Before implantation, ovarian fragments were stitched using non-absorbable sutures (6/0 Prolene; Ethicon, Diegem, Belgium). After 7 days (days 7 and 8 for the left and right sides of the neck, respectively), the animals were anesthetized, and the recovered grafts (Fig 1C–1H) were classified macroscopically by a single operator using a semi-quantitative scoring system (S1 Table). Briefly, the criteria used for the end points were as follows: morphology [1 = necrotic (totally dark graft), 2 = some points of necrosis, 3 = intact and similar to the original graft, 4 = intact and slightly swollen, and 5 = intact and swollen with increased volume]; adhesion of the graft to the host tissue [1 = poor (easy to remove), 2 = slightly inserted, 3 = moderately inserted, 4 = strong (well inserted, with some difficulty in removing), and 5 = intense (very difficult to remove)]; and extent of bleeding at the time of graft removal [1 = absent bleeding, 2 = in up to two areas around the fragment, 3 = in half of the fragment, 4 = in two-thirds of the fragment, and 5 = around the whole fragment].

### Experimental design

In this study, ovarian biopsy fragments (n = 7) from each animal were randomly distributed among the seven treatments (Fig 1B). One ovarian fragment was immediately fixed in 4% paraformaldehyde (Fresh control treatment), while the remaining six fragments (i.e., one fragment for each treatment) were maintained in the holding medium without or with VEGF at 4°C (n = 4) or 25°C (n = 2), being half of the fragments incubated for each temperature with VEGF. The fragments maintained at 4°C were immediately fixed after 24 h of cooling (Non-transplant treatments without or with VEGF) or autografted and retained in the transplant site for 7 days (Transplant 4°C treatments without or with VEGF). Fragments maintained at 25°C were incubated for 30 min and, thereafter, immediately autografted and maintained in the transplant site for 7 days (Transplanted 25°C treatments without or with VEGF). The rationale for harvesting grafted tissues 7 days post-OTT is that profound ischemic injuries and neoangiogenesis occur during the initial phase (i.e., 2–7 days) post-OTT [21, 22]. After incubation or grafting, ovarian fragments from all treatments were fixed for histological analysis. However, only ovarian fragments from the Fresh control and transplanted groups were considered for the immunohistochemistry analyses. Eight replicates of each treatment were performed. Each replicate was composed of one animal that participated in all treatments.

### Blood flow evaluation at the graft site

**Fig 2:**
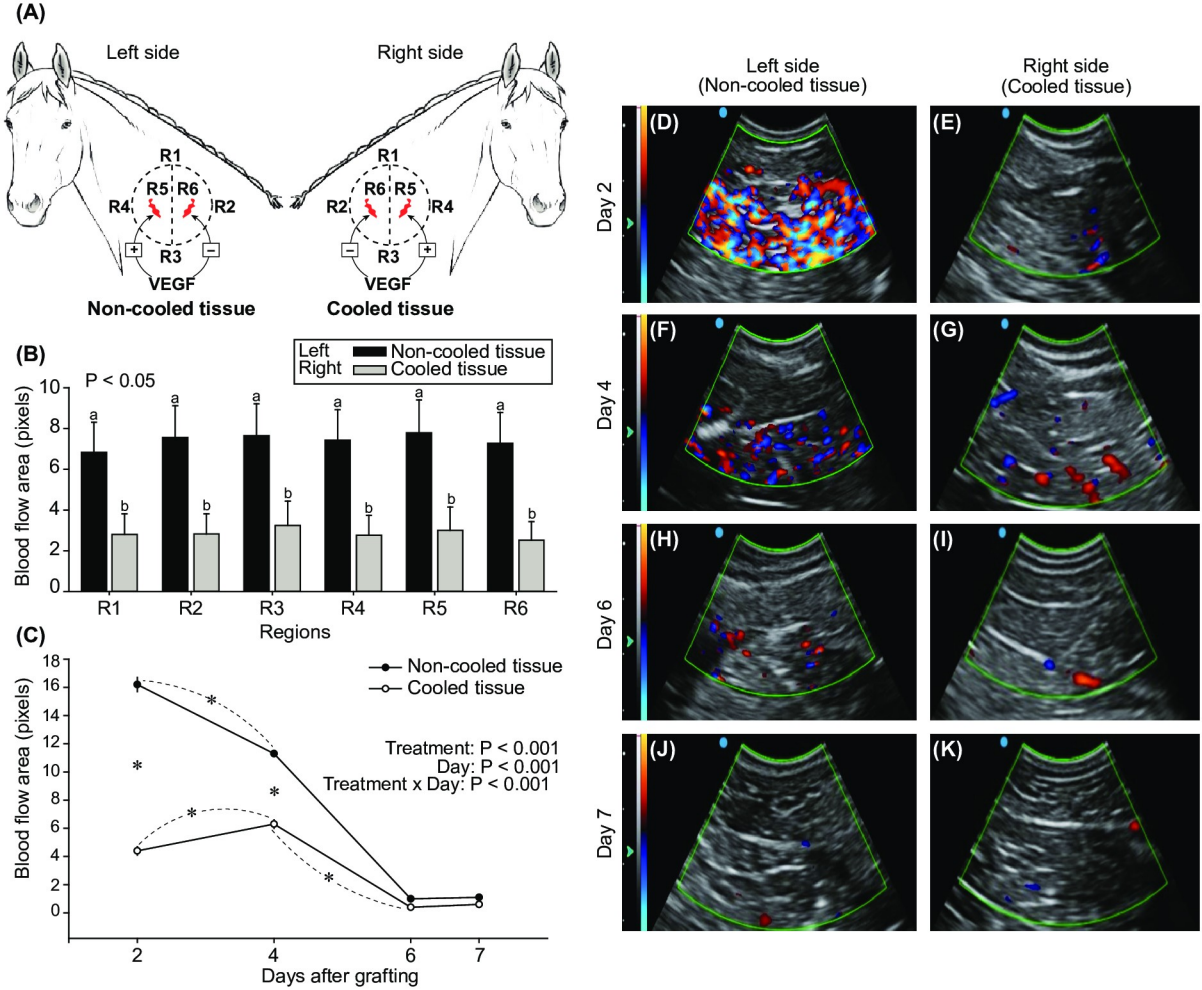
(A) Schematic design of the transplant sites with the different regions (R1, R2, R3, R4, R5, and R6) used to evaluate the local blood flow in each neck side [i.e., left side = non-cooled (25°C) transplant treatments, and right side = cooled (4°C) transplant treatments]. Implanted ovarian tissues, associated or not to previous exposure to VEGF, are shown in red. (B–C) Mean (± SEM; n = 8 animals) blood flow area comparison between non-cooled and cooled fragments (VEGF–/+ combined treatments) at (B) the different regions of the transplant site in each neck side, and (C) different days post-grafting. ^a,b^Within each region, different letters indicate differences between non-cooled and cooled treatments. *Indicates the first significant increase or decrease for each treatment, and a difference between treatments within a day. The probabilities for a treatment effect, day effect, and treatment-by-day interaction are shown. (D–K) Representative color-Doppler ultrasonograms indicating the presence of different degrees of blood perfusion ("hyperemia") in a specific region of the transplant site at days 2, 4, 6, and 7 post-grafting using (D, F, H, J) non-cooled and (E, G, I, K) cooled ovarian tissues. Color signals indicate blood flow in each ultrasonogram.

After the ovarian grafting procedure, blood flow perfusion near the grafted fragments was evaluated using color-Doppler ultrasonography. Ultrasonographic video clips were obtained in four regions (R) surrounding the grafts on the left and right sides of the neck (i.e., R1, R2, R3, and R4) and on the sites of transplantation (R5 left side, Transplant 4°C + VEGF treatment; R5 right side, Transplant 25°C + VEGF treatment; R6 left side, Transplant 4°C treatment; R6 right side, Transplant 25°C treatment). The examinations were performed on days 2, 4, 6, and 7 post-grafting using a portable duplex color-Doppler ultrasound machine (CTS-8800V, SIUI, Jiangsu, China) connected to a microconvex probe (6.5 MHz) (Fig 2A). The distance between regions R1-R3 and R2-R4 was approximately 5 cm. The grafted fragments were located about 2 cm from the scanned areas R1, R2, R3, and R4. During each scanning, three different images with maximum color-Doppler signals of blood flow were obtained from each region and processed for the extraction and quantification of the number of colored pixels as described previously [44]. Then, the ImageJ^®^ software (National Institutes of Health, Millersville, USA), previously calibrated (1 cm = 33 pixels), was used for quantification of the area (cm^2^) of color pixels from each image.

### Histological analysis

All ovarian fragments were fixed in 4% paraformaldehyde for 2 h and then dehydrated in 70% ethanol. After standard histological preparation, the samples were cut into serial sections of 7 μm [45]. Every section was mounted and stained with Periodic Acid-Schiff (PAS) and counterstained with hematoxylin.

### Follicular morphology and development

For follicle morphological evaluation, the histological sections were analyzed using light microscopy (Nikon E200; Tokyo, Japan) at 400× magnification coupled to an image capture system (Nikon, Coolpix 4500). The follicles were classified morphologically as normal (when oocyte nucleus and surrounding granulosa cells are structurally intact) or degenerated (follicles with a retracted cytoplasm, disorganized granulosa cell layers detached from the basement membrane and oocyte with a pyknotic, fragmented, or shrunken nucleus), as previously described [11]. Moreover, the follicles were classified according to the development stage as primordial, oocyte surrounded by a single layer of flattened granulosa cells; developing follicles such as transitional, a single layer of both flattened and cuboidal granulosa cells around the oocyte; primary, a single layer of cuboidal granulosa cells around the oocyte; and secondary, oocyte with zona pellucida surrounded by two or more layers of cuboidal granulosa cells [10]. Follicle development (i.e., quiescent primordial follicles potentially activated and developed to transitional, primary, and secondary-stage follicles) was calculated by considering the number of normal developing follicles divided by the total number of normal preantral follicles multiplied by 100). All histological sections were examined by the same operator who was unaware of mare identity and treatment. To avoid double counting, only preantral follicles containing oocytes with a visible nucleus were analyzed.

### Follicular and stromal cell densities

Intracellular interactions between follicles and stromal cell factors are required to regulate follicle growth and oocyte maturation [46]. Therefore, in this study, the follicular and stromal cell densities were investigated in all treatments. The follicular density was determined as previously reported [45]. Briefly, 10% of all histological sections of each treatment were delimited using image editing software (ImageJ), and the area was recorded after scale calibration. Thereafter, the follicular density was calculated by dividing the number of normal preantral follicles by the area of the ovarian section (cm^2^). For evaluation of the ovarian stromal cell density, a total of 10% of all histological sections of each treatment were analyzed as described by Alves et al. [17]. Four random fields (50 × 50 μm = 2,500 μm^2^) per selected section were recorded to calculate the mean stromal cell density per ovarian fragment.

### New vessel density (CD31) and stromal cell apoptosis (caspase-3)

Immunolocalizations of cluster of differentiation 31 (CD31) and caspase-3 were performed to evaluate the presence of new blood vessels [47] and stromal cell apoptosis (adapted from [48]), respectively. The ovarian fragment sections were mounted as for classical histology and serially sectioned into 5-μm thickness in positively charged slides. Subsequently, after the epitopes were activated by incubation at 98°C for 5 min in low PH buffer (DM831, DAKO, Carpinteria, CA, USA), the blocking endogenous peroxidase activity was performed with 3% H2O2 diluted in methanol. After that, the sections were incubated for 30 min in a humid chamber at room temperature with rabbit polyclonal to CD31 (1:100; AB28364, Abcam, Cambridge, UK) and rabbit polyclonal to caspase-3 (1:100; AB4051, ABCAM) primary antibody, and subsequently for 30 min with the biotinylated goat anti-rabbit IgG secondary antibody (1:200; AB97049, ABCAM). Then, the slides were incubated for 30 min with avidin-biotin (Vector Laboratories, Burlingame, CA, USA), and the immunoreaction was visualized through the use of diaminobenzidine (DAB, ACB030; ScyTek Laboratories Inc., West Logan, USA). Finally, the sections were counterstained with hematoxylin and Scott’s solution. Negative controls were performed by not using the primary antibody. Healthy and intoxicated kidney tissue sections were used as positive controls for CD31 and caspase-3, respectively. To calculate the density of new vessels, the CD31-stained vessels were counted in two different sections in each tissue sample, and the average of the number of stained blood vessels per cm^2^ was determined. The caspase-3 was used to detect stromal cell apoptosis by counting positive cells in random areas (100 × 100 μm = 10,000 μm^2^). Immunostaining was evaluated based on images obtained at high magnification (400×) and analyzed using ImageJ software.

### AgNOR staining

To investigate the stromal cell proliferation index, AgNOR staining was used to quantify the number of argyrophilic nucleolar organizing regions (NOR) that have silver (Ag) affinity for the stromal cell nucleus. For this purpose, ovarian tissue fragments from the control and autotransplanted treatments were histologically processed, as described earlier. Thereafter, NOR labeling was performed and interpreted using an adapted protocol previously reported [49]. Briefly, after reduction with potassium iodide 1%, slides containing ovarian tissue sections of 5 μm (5 sections per ovary/per treatment) were stained with 50% silver nitrate solution in a colloid solution (2:1) in a darkroom and counterstained with 0.1% safranin. To quantify the NORs, five sections in each slide were examined under ×100 oil immersion objective, and three random fields (50 × 50 μm = 2,500 μm^2^) per selected section were recorded. NORs of all the nuclei of visible stromal cells were counted.

### Collagen types I and III fibers in ovarian tissue

Several physiological events, such as revascularization, fibrosis, repair to damages, and cell death have been associated with collagen fibers type I and III [50]. To evaluate the collagen density in selected treatments, relative areas of fibrosis (rich collagen deposits) were evaluated [23]. Briefly, four histological sections of each ovarian fragment per treatment were processed for classical histology and stained using standard protocol by the Picrosirius red stain (0.1%) (#365548-5G, Sigma-Aldrich, Switzerland) with saturated Picric acid solution (1.2%) for 1 h at room temperature. Polarizing microscopy (Nikon E200; Tokyo, Japan) at ×400 magnification coupled to an image capture system (Nikon, Coolpix 4500) was used. The total collagen fiber content in the connective tissue and the different types of collagen fibers based on polarizing colors (i.e., collagen type I fibers stained yellow with orange birefringence and collagen type III fibers stained with green birefringence) were analyzed as previously described [51]. Images were analyzed by red, green, and blue threshold measurement to obtain the percentage of red and green colors (expressed in pixels) and 3D data representation of the pixels for each image using ImageJ software [44]. The blue color representing all the other cellular types was omitted.

### Statistical analyses

The statistical analyses were performed using Sigma Plot version 11.0 (Systat Software Inc., USA). The normality (Shapiro-Wilk test) and homogeneity of variance (Levene’s test) were initially evaluated. One-way ANOVA, followed by post-hoc test, was used to detect differences between the Fresh control and the other treatments. Two-way ANOVA was used for comparisons between the non-transplant and transplant treatments and to test for the main effects of treatment under similar or different temperatures (4°C or 25°C) and VEGF (–or +) exposure. The area of colored pixels (blood-flow) was compared between the left and right sides within the same region and days after grafting using the Mann-Whitney test. Moreover, blood flow area comparison was evaluated within the same treatment at different days by repeated-measures ANOVA on ranks. The Spearman correlation test was used to verify the association between the parameters analyzed. Data are presented as mean (± SEM). Differences were considered significant at *P* < 0.05 (two-sided), and values > 0.05 and < 0.1 indicate that a difference approached significance.

## Results

### Ovarian graft appearance and blood flow at the graft site

The ovarian tissue transplantation and harvesting procedures took, on average, 11 ± 0.5 min (range, 8–16 min) and 24 ± 0.9 min (range, 17–29 min) to perform, respectively. All grafted ovarian fragments were successfully recovered after transplantation. Overall, the macroscopic appearance of the grafted fragments immediately after harvesting (Fig 1E–1H), according to morphology, adherence, and bleeding, was similar among treatments (S1 Table). Despite the region of the neck (Fig 2A), the overall blood flow between days 2 and 7 post-grafting was lower (*P* < 0.001) on the right side of the neck where fresh cooled fragments (i.e., Transplant 4°C and Transplant 4°C + VEGF treatments combined) were used for grafting (Fig 2B). The cooling process of the fragments reduced the acute hyperemia (i.e., blood flow at the graft site) during the first 4 days post-grafting (Fig 2C); thereafter, the blood flow tissue perfusion was similarly reduced to minimum levels in both cooled and non-cooled transplanted treatments. Representative color-Doppler ultrasonograms with color pixels indicating blood flow in the left (Fig 2D, 2F, 2H and 2J) and right (Fig 2E, 2G, 2I and 2K) sides of the neck on days 2, 4, 6, and 7 are shown.

### Follicular morphology and development

**Fig 3:**
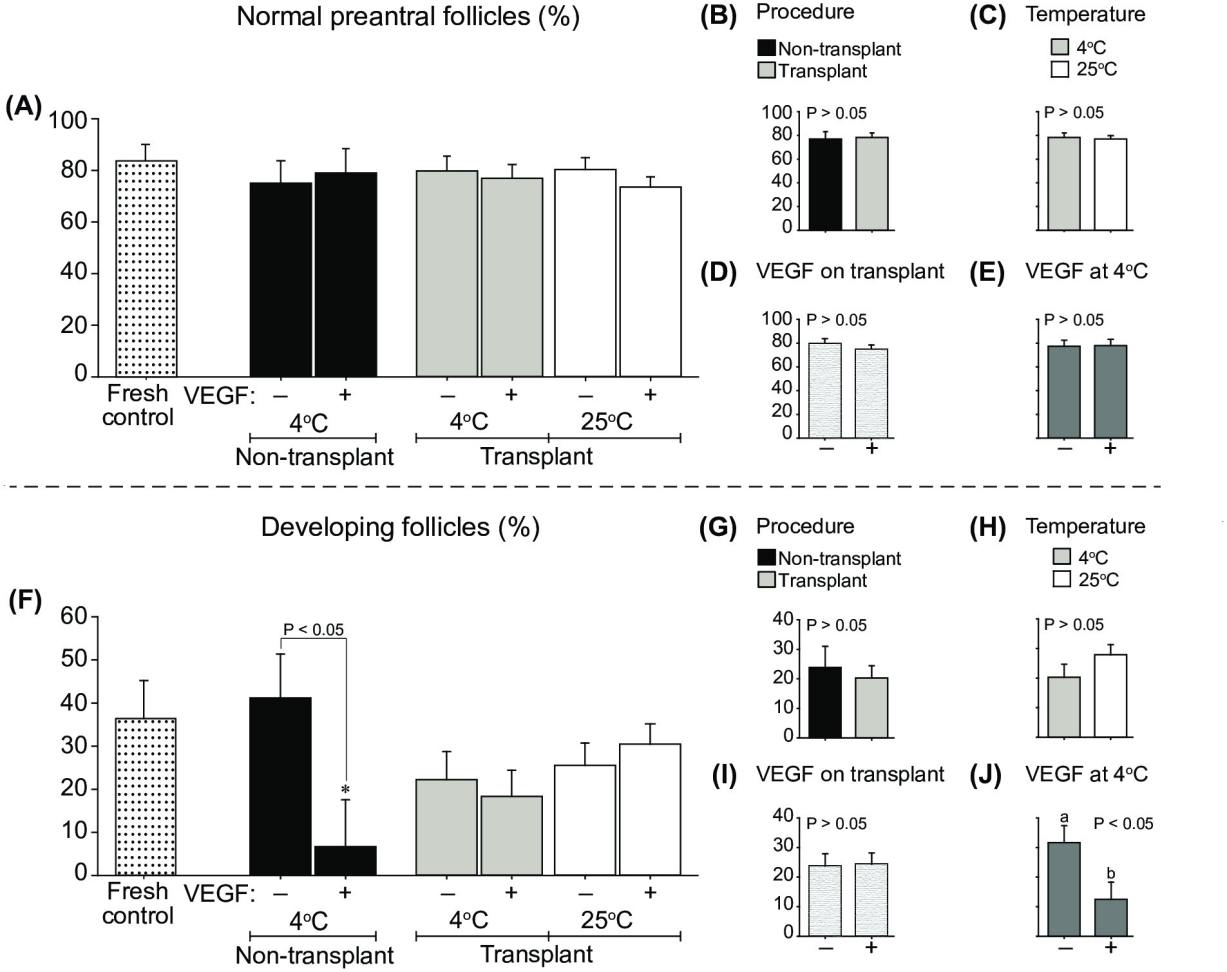
Mean (± SEM; n = 8 animals) percentages of (A–E) morphologically normal preantral follicles (i.e., primordial to secondary-stage follicles) and (F–J) developing follicles (i.e., normal developing follicles divided by the total number of normal preantral follicles multiplied by 100) after 7 days of heterotopic ovarian tissue autotransplantation in mares. (A and F) Comparisons of Fresh control with other treatments (one-way ANOVA) and (A–J) between non-transplant and transplant treatments (two-way ANOVA) were made. The effects of (B and G) procedure (non-transplant *vs*. transplant) using cooled tissue, (C and H) cooling/temperature (transplants at 4°C *vs*. 25°C) regardless of VEGF, (D and I) VEGF on transplant regardless of temperature, and (E and J) VEGF on cooled ovarian tissue were evaluated after grafting. *Indicates a difference (*P* < 0.05) between the Fresh control and another group. ^a,b^Within any effect analyzed, different letters indicate differences between treatments. P-values for the main analyses are shown.

A total of 442 preantral follicles were recorded in 15,965 histological sections analyzed from 56 biopsy tissue samples (i.e., seven fragments x eight mares). The percentage of normal preantral follicles did not differ between the Fresh control and the other treatments (range, 73.5 ± 4.1 to 83.8 ± 6.2 normal follicles; one-way ANOVA; Fig 3A). Using a two-way ANOVA to compare the treated groups, the percentage of normal follicles in cooled fragments was not influenced by the transplant procedure (i.e., Non-transplant *vs*. Transplant 4°C treatments; Fig 3B), the temperature of treated fragments (Transplant 4°C *vs*. Transplant 25°C; Fig 3C), the VEGF on transplant treatments (Fig 3D), or the VEGF on cooled fragments (Fig 3E). The percentages of developing follicles were similar between the Fresh control and transplant treatments (Fig 3F). However, the percentage of developing follicles was lower (*P* < 0.05) in the Non-transplant + VEGF treatment than in the Fresh control group and the Non-transplant treatment. Finally, although the percentage of developing follicles was not influenced by the effect of transplant (Fig 3G), the temperature on transplant treatments (Fig 3H), or by the VEGF on transplant treatments (Fig 3I), a reduction (*P* < 0.05) of developing follicles was observed in cooled fragments exposed to VEGF (Fig 3J).

### Follicular and stromal cell densities

**Fig 4:**
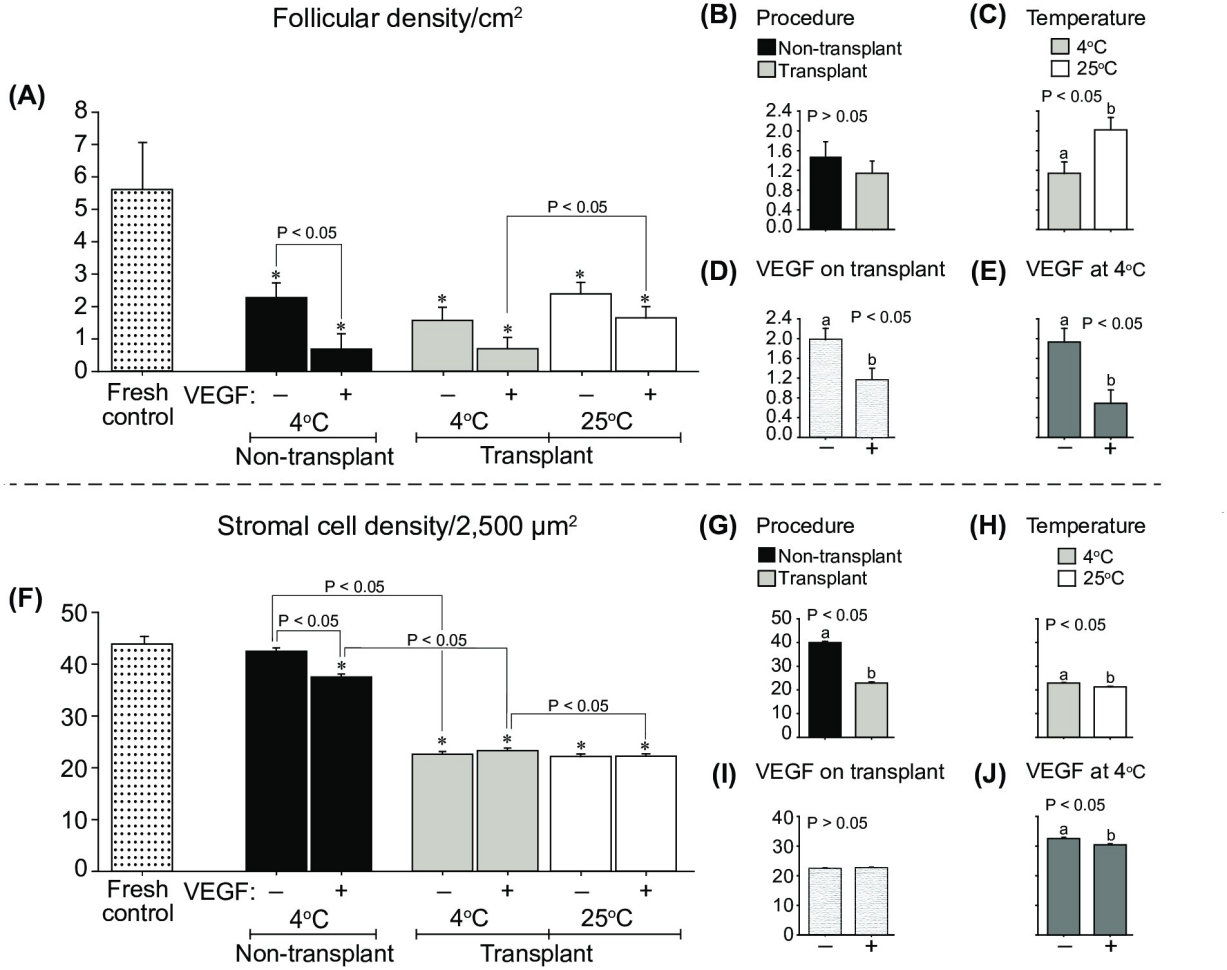
Mean (± SEM; n = 8 animals) percentage of (A–E) follicular density and (F–J) stromal cell density after 7 days of heterotopic ovarian tissue autotransplantation in mares. (A and F) Comparisons of Fresh control with other treatments (one-way ANOVA) and (A–J) between non-transplant and transplant treatments (two-way ANOVA) were made. The effects of (B and G) procedure (non-transplant *vs*. transplant) using cooled tissue, (C and H) cooling/temperature (transplants at 4°C *vs*. 25°C) regardless of VEGF, (D and I) VEGF on transplant regardless of temperature, and (E and J) VEGF on cooled ovarian tissue were evaluated after grafting. *Indicates a difference (*P* < 0.05) between the Fresh control and another group. ^a,b^Within any effect analyzed, different letters indicate differences between treatments. P-values for the main analyses are shown.

The follicular density/2,500 μm^2^ in the Fresh control group was higher (*P* < 0.05) than in all other treated groups (Fig 4A). In the Non-transplant treatments, fragments exposed to VEGF had a lower (*P* < 0.05) follicular density. Moreover, when comparing transplant treatments exposed to VEGF, cooled fragments had lower (*P* < 0.05) follicular density than fragments kept at room temperature. Although the follicular density in cooled fragments was not influenced by the effect of the transplant (Fig 4B), the follicular density in room-temperature-transplanted fragments was higher (*P* < 0.05) than in cooled fragments (Fig 4C). When transplant treatments at different temperatures were combined (Fig 4D), ovarian fragments exposed to VEGF had a lower (*P* < 0.05) follicular density. Likewise, cooled fragments exposed to VEGF (Non-transplant and Transplant treatments combined) had lower (*P* < 0.05) follicular density than the fragments nonexposed to VEGF (Fig 4E).

The stromal cell density/2500 μm^2^ did not differ between the Fresh control and the Non-transplant cooled treatment (Fig 4F). However, lower (*P* < 0.05) stromal cell density was observed in the Non-transplant + VEGF cooled treatment and all the transplant treatments compared with the Fresh control. In the Non-transplant treatments, fragments exposed to VEGF had a lower (*P* < 0.05) stromal cell density. However, regardless of the exposure to VEGF, cooled fragments submitted to a grafting procedure (Transplant 4°C treatments) had a lower (*P* < 0.05) stromal cell density than the Non-transplant cooled treatments (Fig 4F and 4G). In contrast, the exposure to VEGF resulted in a higher (*P* < 0.05) stromal cell density in cool-transplanted fragments (Transplant 4°C + VEGF treatment) compared to fragments kept at room temperature before grafting (Transplant 25°C + VEGF treatment). Thus, when treatments were combined, the overall stromal cell density after grafting was higher (*P* < 0.05) in cooled than in non-cooled fragments (Fig 4H), whereas it was not affected by exposure to VEGF (Fig 4I). In contrast, cooled fragments exposed to VEGF (Non-transplant and Transplant treatments combined) had lower (*P* < 0.05) stromal cell density than the fragments nonexposed to VEGF (Fig 4J).

### New vessel density (CD31), stromal cell apoptosis (caspase-3) and proliferation index (AgNOR)

**Fig 5:**
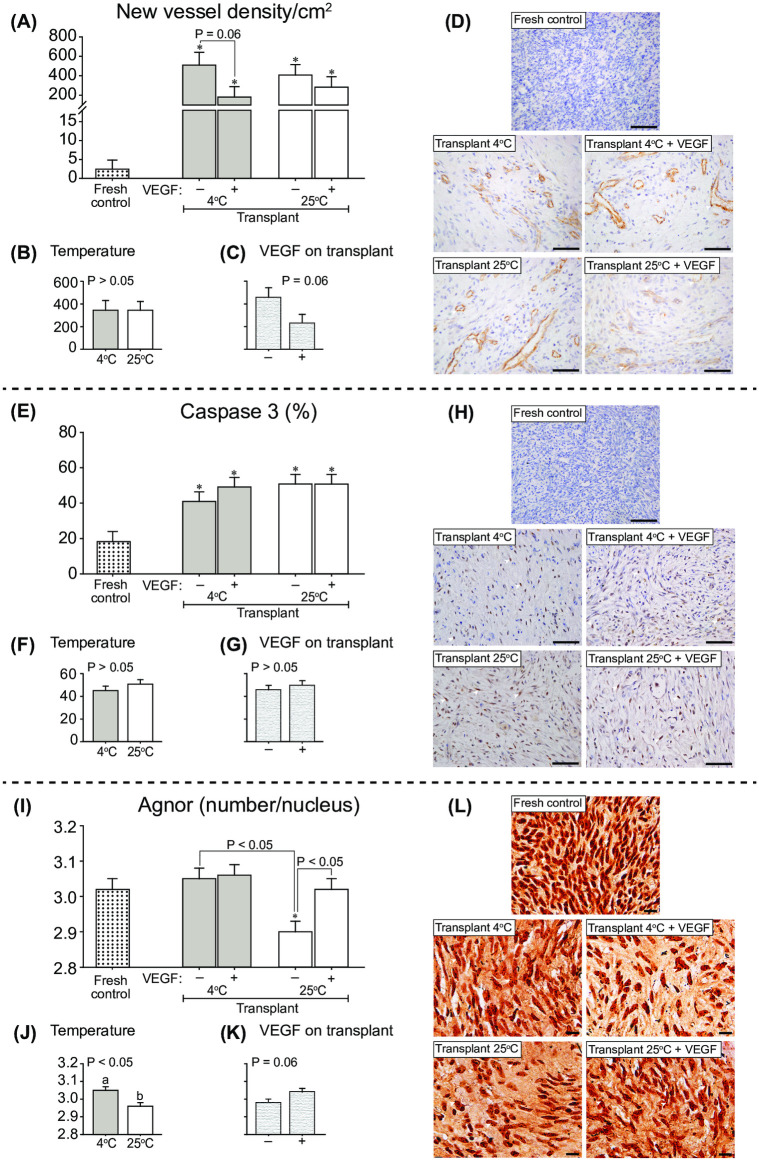
Mean (± SEM; n = 8 animals) (A–C) new vessel density, (E–G) percentage of stromal cells labeled with caspase 3, and (I–K) percentage of nucleolus organizer regions (NORs) per nucleus of stromal cells after 7 days of heterotopic ovarian tissue autotransplantation in mares. (A, E, I) Comparisons of Fresh control with other treatments (one-way ANOVA) and between transplant treatments within different conditions (two-way ANOVA) were made. The effects of (B, F, J) cooling/temperature (transplants at 4°C *vs*. 25°C) regardless of VEGF, and (C, G, K) VEGF on transplant regardless of temperature were evaluated after grafting. *Indicates a difference (*P* < 0.05) between the Fresh control and another group. ^a,b^Within any effect analyzed, different letters indicate differences between treatments. P-values for the main analyses are shown. Representative histological images with (D) the presence of CD31-stained new vessels, (H) stromal cells labeled with caspase 3, and (L) NORs in stromal cell nucleus are shown (arrowheads) for the Fresh control and transplant treatments. (D and H) Magnification = 400×, scale bars = 20 μm; and (L) Magnification = 1000×, scale bar = 10 μm.

The density of new vessels increased (*P* < 0.05) in the transplant treatments compared with the Fresh control treatment (Fig 5A). In cooled fragments, the vessel density tended (*P* = 0.06) to be lower in fragments exposed to VEGF (Transplant 4°C + VEGF treatment). Although the vessel density was not affected by the temperature at which the fragments were maintained before grafting (Fig 5B), when fragments were exposed to VEGF, a lower (*P* = 0.06) vessel density was observed (Fig 5C). The presence of vessels in representative histological slides is shown for the Fresh control and transplant treatments (Fig 5D).

The presence of stromal cell apoptosis, evaluated by immunolocalization of the caspase-3 protein (Fig 5H), was higher (*P* < 0.05) in all transplant treatments compared to the Fresh control treatment (Fig 5E). The percentage of stromal cell apoptosis was not affected by either the temperature (Fig 5F) or the exposure of the fragments to VEGF before grafting (Fig 5G).

When the stromal cell proliferation index was estimated by using AgNOR, the number of NORs per stromal cell nucleus (Fig 5L) was lower (*P* < 0.05) in the Transplant 25°C treatment when compared with the Fresh control and the other transplant treatments, except for the Transplant 4°C + VEGF treatment (Fig 5I). The number of NORs was higher (*P* < 0.05) in cooled treatments (Fig 5J) and tended (*P* = 0.06) to be higher in treatments exposed to VEGF (Fig 5K).

### Quantification of fibrosis (collagen types I and III fibers)

**Fig 6:**
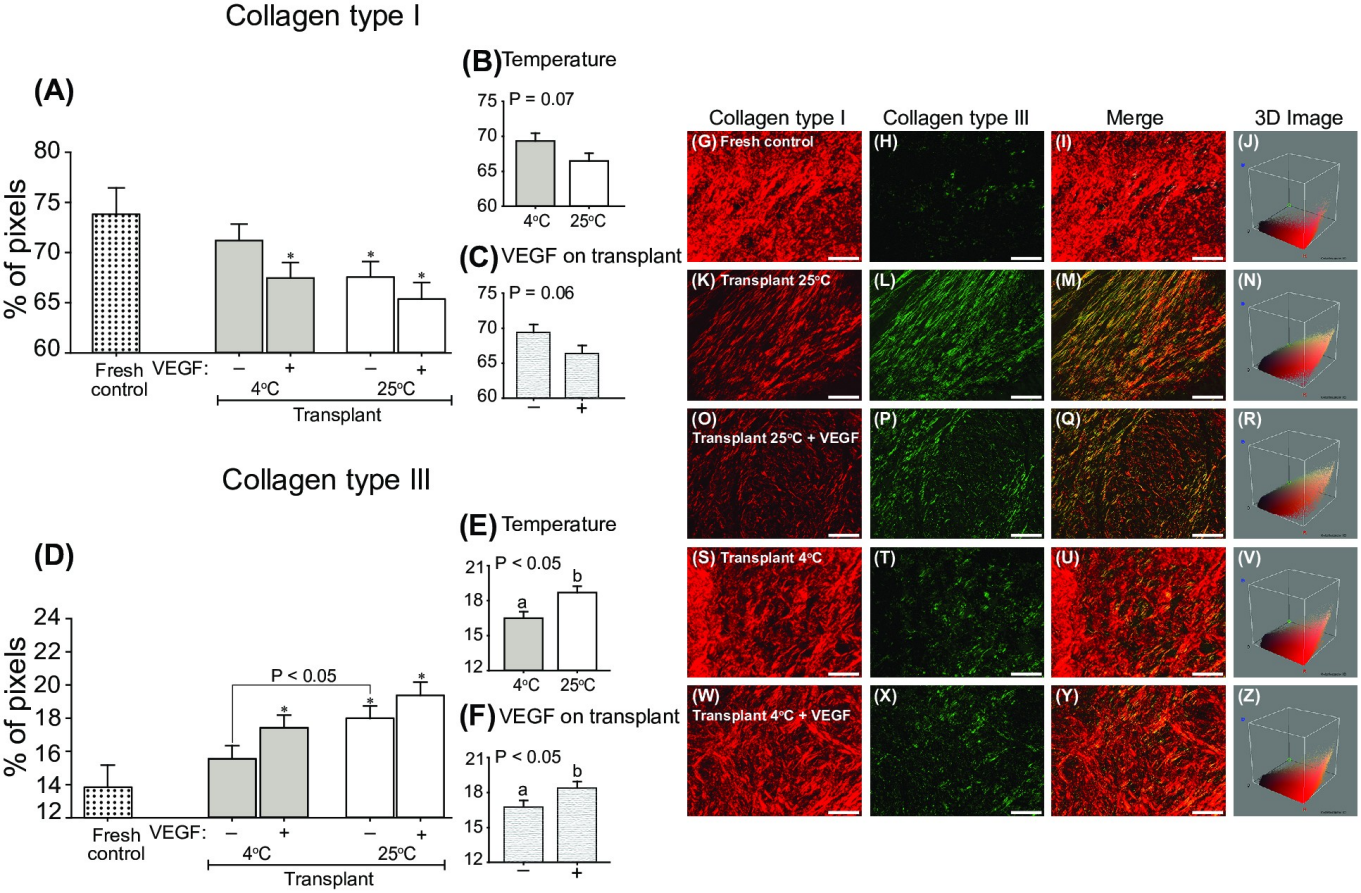
Mean (± SEM; n = 8 animals) collagen type (A–C) I and (D–F) III fibers after 7 days of heterotopic ovarian tissue autotransplantation in mares. (A and D) Comparisons of Fresh control with other treatments (one-way ANOVA) and between transplant treatments within different conditions (two-way ANOVA) were made. The effects of (B and E) cooling/temperature (transplants at 4°C *vs*. 25°C) regardless of VEGF, and (C and F) VEGF on transplant regardless of temperature were evaluated after grafting. *Indicates a difference (*P* < 0.05) between the Fresh control and another group. ^a,b^Within any effect analyzed, different letters indicate differences between treatments. P-values for the main analyses are shown. Representative histological images of ovarian tissue stained with Picrosirius red stain showing collagen type (G, K, O, S, W) I and (H, L, P, T, X) III fibers, and (I, M, Q, U, Y) merged images are depicted for the Fresh control and transplant treatments. (J, N, R, V, Z) 3D images illustrate the spatial distribution of the two types of collagen in each group. Image magnification = 400×; scale bars = 20 μm.

The percentage of pixels regarding collagen type I and III fibers evaluated in ovarian tissue fragments (Fig 6A–6F) and representative histological and 3D images (Fig 6G–6Z) are shown. The percentage of collagen type I was lower (*P* < 0.05) in transplanted treatments compared with the Fresh control treatment, except for the Transplant 4°C treatment (Fig 6A). The percentage of collagen type I fiber tended (*P* = 0.06–0.07) to be higher in cooled treatments (Fig 6B) and lower in treatments exposed to VEGF (Fig 6C). In contrast to what was observed for collagen type I, the percentage of collagen type III was higher (*P* < 0.05) in transplanted treatments compared with the Fresh control treatment, except for the Transplant 4°C treatment (Fig 6D). Furthermore, cooled fragments nonexposed to VEGF had a lower (*P* < 0.05) percentage of collagen type III in the Transplant 25°C than in the Transplant 4°C treatment. The percentage of collagen type III fibers was lower (*P* < 0.05) in cooled treatments (Fig 6E) and higher (*P* < 0.05) in fragments exposed to VEGF (Fig 6F).

### Correlations between end points

Correlation analyses between several studied end points are shown (S1 Fig). A negative correlation (*P* < 0.05) was observed between normal follicles and caspase 3 (S1D Fig). Statistical tendencies (*P* = 0.07–0.09) for a difference were observed for positive correlations between developing follicles and blood flow, collagen type III and blood flow, follicular density and vessel density (S1A, S1B and S1E Fig), and for negative correlations between AgNOR and caspase 3, and collagen type I and AgNOR (S1C and S1F Fig).

## Discussion

This is the first study on ovarian tissue transplantation/grafting in horses. Herein we investigated the efficiency of a heterotopic ovarian tissue autotransplantation technique by testing the effects of hypothermia (i.e., cooling at 4°C *vs*. room temperature at 25°C) and exposure to VEGF (–/+) on ovarian biopsy fragments before grafting.

The site of the transplant (neck) and the subcutaneous implantation of the ovarian fragments chosen for this study were well accepted by the animals and preserved the integrity of the surgical wound until graft recovery. The surgical procedures of transplant and harvesting were relatively simple and quickly performed but did require a well-trained operator. The subcutaneous site has been used previously for grafting of ovarian tissue in several species (mice [52, 53], goats [44], non-human primates [23], and humans [54, 55]). A temporary inflammatory reaction is usually expected to occur at the transplantation site during the first days post-grafting. In the present study, after 7 days of grafting, the macroscopic appearance of the implanted fragments immediately after harvesting seemed to be normal and was similar among treatments. Our macroscopic findings for the grafted tissue (i.e., morphology, adherence, and bleeding) were also similar to those previously reported for human tissue after 7 days post-grafting in a subcutaneous site [23, 44]. In this regard, our research group has recently reported lesser tissue hemorrhage after harvesting grafted ovarian fragments from a subcutaneous than from an intramuscular site in goats [44].

To further support the efficiency of the grafting procedure, the data of the present study demonstrate that, even 7 days after transplant, the percentage of normal and developing preantral follicles in cooled or room-temperature-exposed fragments was similar to that of the Fresh control treatment. On the other hand, in the present study, when compared with the Fresh control, the transplanted fragments had lower follicular and stromal cell densities and collagen type I fibers but higher vessel density, apoptotic stromal cells, and collagen type III fibers. Some studies suggest that after OTT, an increase in growing follicles occurs concurrently with a significant depletion in primordial follicles [56]. This follicle loss (i.e., depletion in primordial follicle density) during the first week post-transplant could be explained by a potential increased follicle activation [56], damage due to cryopreservation, or stress-induced factors [57–59]. A profound ovarian stromal cell loss has been reported to occur after grafting in several species (mouse [60], rat [61], monkey [62], goat [47], and human [29]) as well as after *in vitro* culture (goat [63]) and ovarian tissue storage/transport (mare [13]). Although satisfactory results have been shown after OTT in women [1], the mechanisms involved in ovarian graft viability are not well understood. The OTT technique usually leads to marked follicle losses [64], decreased stromal cell numbers [22] and tissue viability [56], and increased tissue apoptosis and fibrosis [23]; these outcomes are usually caused by ischemic injuries, hormonal and molecular interactions, cryodamage [25], prolonged hypoxia [65] and oxidative stress [66].

Other remarkable findings in this study were a negative correlation between the percentage of morphologically normal follicles and apoptotic stromal cells labeled with caspase 3, in addition to higher and lower percentages of collagen type I and III fibers, respectively, in cooled fragments. In soft tissues, collagen type I and III fibers are the main structural components (70% type I and 20% type III [50]). Collagen type I fibers are important in *in vitro* and *in vivo* angiogenesis [67] because, in association with fibrin, they modulate collagen-fibrin microtissues to promote vessel formation [68]. Contrarily, collagen type III fibers increase the deposition of extracellular matrix remodeling for wound repair and fibrosis [69]. Therefore, the data of the present study supported the idea that the collagen matrix composition was altered by potentially activating the mechanisms controlling tissue development, remodeling, and regeneration in response to stress induced by the engrafting surgical procedure. The findings herein described may contribute to a greater understanding of tissue regeneration and remodeling after a grafting process.

In non-transplanted cooled ovarian tissue, even though the percentage of follicle survival (normal follicles) was not affected by VEGF exposure, the percentages of developing follicles and follicular and stromal cell densities were negatively affected by exposure to VEGF. Regarding grafted ovarian tissue, VEGF exposure had a negative effect on follicular and vessel densities and reduced the collagen type I fibers. Nonetheless, grafted ovarian tissue pre-exposed to VEGF had a higher proportion of collagen type III fibers and a greater number of NORs. Several *in vitro* and *in vivo* studies have shown that the effects of VEGF on cell survival and proliferation have produced contradictory results [4, 37, 70]. In this regard, *in vivo* studies in mice and sheep have shown that the use of VEGF induced new blood vessel formation and prevented follicular loss after ovarian tissue transplantation [26, 28]. Moreover, the direct administration of VEGF into the rat ovary increased vascularization [71] and the number of primary, secondary and antral follicles [72], and reduced apoptosis [73]. In contrast, higher doses of VEGF (500 ng/ml) administered in rats decreased neuronal cell survival and mRNA expression of the anti-apoptotic protein Bcl-2 and increased the apoptotic protein caspase 3 [74]. In the present study, the deleterious effect of VEGF on developing follicles, follicular and stromal cell and vessel densities, and collagen type I was more likely to be due to the prolonged exposure time (24 h) to VEGF. This assumption is supported by the following findings: (i) the exposure of fragments to VEGF at room temperature for a short period (30 min) did not affect the studied end points, except for a higher number of AgNORs, and (ii) the dose of VEGF herein used has not revealed any toxic effect in studies *in vitro* (goats [70] and cattle [75]) and *in vivo* (mice [72], rats [72], rabbits [29], cattle [76], and humans [37]). In regard to angiogenesis, an improper balance between stimulators and inhibitors can lead to excessive angiogenic signaling and later pathological processes, including intense endothelial permeability that leads to vasogenic edema, inflammatory process, apoptosis, and cell death [77–80]. In the present study, the cooling process seemed to minimize the acute inflammatory (hyperemia) response post-grafting. Also, the cooling process for 24 h without VEGF did not jeopardize the quality of the ovarian fragments before grafting. With this in mind, it is worth testing the effect of VEGF exposure during shorter incubation times but keeping the ovarian tissue cooled for similar/longer periods (i.e., 12–24 h). This approach would make practical in the future the transportation of ovarian biopsied fragments to specialized reproductive centers and the spread of the OTT technique to species of genetic and economic interest [13, 81, 82].

Lower temperatures have been shown to be advantageous for transportation of ovarian fragments to be used for several purposes in different species [40, 57, 83, 84]. The present work shows that even after a 24 h cooling process before grafting, the percentages of normal and developing follicles, the vessel density/cm^2^ (CD-31), and the proportion of apoptotic stromal cells labeled with caspase 3 were similar to those of transplanted fragments previously kept at room temperature for 30 min. Moreover, after transplantation, cooled fragments had a higher stromal cell density and proliferation (AgNOR) as well as a greater amount of collagen type I fibers. However, cooled ovarian fragments had lower blood flow at the grafting site as well as a smaller proportion of collagen type III fibers and follicular density post-grafting. Herein, the use of color-Doppler ultrasonography was used as a novel non-invasive technique for monitoring the local vascular perfusion on superficial grafting sites in horses. A similar approach has been used recently by our team to monitor local blood flow on superficial ovarian grafts in goats [44]. Although lower blood flow at the transplant site was observed for 4 days after cooled ovarian fragments were grafted, local blood flow decreased to basal levels 6 days after grafting, becoming similar to that of fragments kept at room temperature. The higher blood flow observed at the grafting site in all transplanted groups in the first 4 days was potentially due to a transient local inflammatory response, as previously discussed [85]. In the present study, the lower follicular density observed in cooled fragments post-grafting was potentially due to the ischemia that occurs immediately after transplantation. Studies have demonstrated that ischemia-reperfusion and hypoxia play a major role in follicle depletion during the first days (approximately 1 week) after transplantation [44, 47, 86]. Altogether, the results of the present study showed that the efficiency of the cooling procedure in preserving follicular and stromal cell quality was comparable to that of fragments maintained for a short period at room temperature before transplantation. These results are in agreement with the concept that ovarian tissue subjected to hypothermic storage (4°C) has a decreased cellular metabolism and oxygen requirement [87, 88], which prevents mitochondrial enzymatic activity [87], thus avoiding a potential follicular activation and tissue autolysis [42]. Hence, the cooling procedure has been widely used during the transportation of ovarian tissue and has produced satisfactory rates of follicle survival in different species (for review, see [89]).

In conclusion, this is a novel study on ovarian tissue transplantation in a large livestock species (i.e., horses). Overall, the heterotopic ovarian tissue autotransplantation technique herein used, under our experimental conditions, did not lead to improved results when VEGF was added to the holding medium before grafting. The cooling procedure of ovarian fragments before grafting did reduce the blood flow transiently at the site of transplantation. Despite that result, this study shows that the cooling procedure of at least 24 h can be successfully used for the preservation of ovarian tissue quality before grafting since it was able to maintain satisfactory rates of follicle survival and development, stromal cell survival and proliferation, and vessel density. Therefore, cooling ovarian fragments before grafting can be a useful approach for optimizing the quality of ovarian tissue specimens during transportation to specialized reproductive centers. This approach could, therefore, facilitate the transport of ovarian specimens, making the OTT technique more feasible for use in preserving/improving the fertility of mares and enhancing its potential for application to other profitable livestock and endangered species.

## Supporting information

S1 Table Mean (± SEM; n = 8 animals) macroscopic end points of grafts evaluated 7 days after heterotopic autotransplantation. (DOCX)

S1 Fig Spearman correlation (r) analyses between end points after 7 days of heterotopic autotransplantation in mares. (A) Developing follicles and (B) collagen type III fibers versus blood flow area; (C) number of AgNORs and (C) percentage of morphologically normal follicles versus caspase 3; (E) follicular density versus vascular density; and (F) collagen type I fibers versus the number of AgNORs. Each circle on the chart represents an ovarian fragment evaluated.
(PDF)
