# Correction: Heterotopic autotransplantation of ovarian tissue in a large animal model: Effects of cooling and VEGF

**DOI:** 10.1371/journal.pone.0252699

**Published:** 2021-05-28

**Authors:** The PLOS ONE Staff

The eighth author’s name is spelled incorrectly. The correct name is: José R. Figueiredo. The publisher apologizes for the error.
