# Supplementary material for: Heterotopic autotransplantation of ovarian tissue in a large animal model: Effects of cooling and VEGF
Source: PLoS One. 2020 Nov 4;15(11):e0241442. doi: 10.1371/journal.pone.0241442 (PMC7641372; doi:10.1371/journal.pone.0241442)
Supplement: S1 Table — (DOCX) [file pone.0241442.s001.docx]

**S1 Table Mean (± SEM; n = 8 animals) macroscopic end points of grafts evaluated 7 days after heterotopic autotransplantation.**

| Macroscopic end points | | | |
| --- | --- | --- | --- |
| Treatments | Morphology^†^ | Adherence^‡^ | Bleeding^§^ |
| Transplant 4°C | 4.3 ± 0.1 | 3.7 ± 0.3 | 2.5 ± 0.2 |
| Transplant 4°C + VEGF | 4.3 ± 0.3 | 3.7 ± 0.2 | 2.2 ± 0.3 |
| Transplant 25°C  Transplant 25°C + VEGF | 4.2 ± 0.2  3.6 ± 0.3 | 3.6 ± 0.1  3.7 ± 0.3 | 2.5 ± 0.2  3.1 ± 0.3 |

^†^Morphological classification: 1 = necrotic (totally dark graft), 2 = some points of necrosis, 3 = intact and similar to the original graft, 4 = intact and slightly swollen, and 5 = intact and swollen with increased volume; ^‡^Adhesion of the graft to the host tissue: 1 = poor (easy to remove), 2 = slightly inserted, 3 = moderately inserted, 4 = strong (well inserted, with some difficulty in removing), and 5 = intense (very difficult to remove); ^§^Extent of bleeding at the time of graft removal: 1 = absent bleeding, 2 = in up to two areas around the fragment, 3 = in half of the fragment, 4 = in two-thirds of the fragment, and 5 = around the whole fragment. No statistical difference was observed among treatments for any of the end points.
