# Supplementary figures and images for: Heterotopic autotransplantation of ovarian tissue in a large animal model: Effects of cooling and VEGF
Source: PLoS One. 2020 Nov 4;15(11):e0241442. doi: 10.1371/journal.pone.0241442 (PMC7641372; doi:10.1371/journal.pone.0241442)

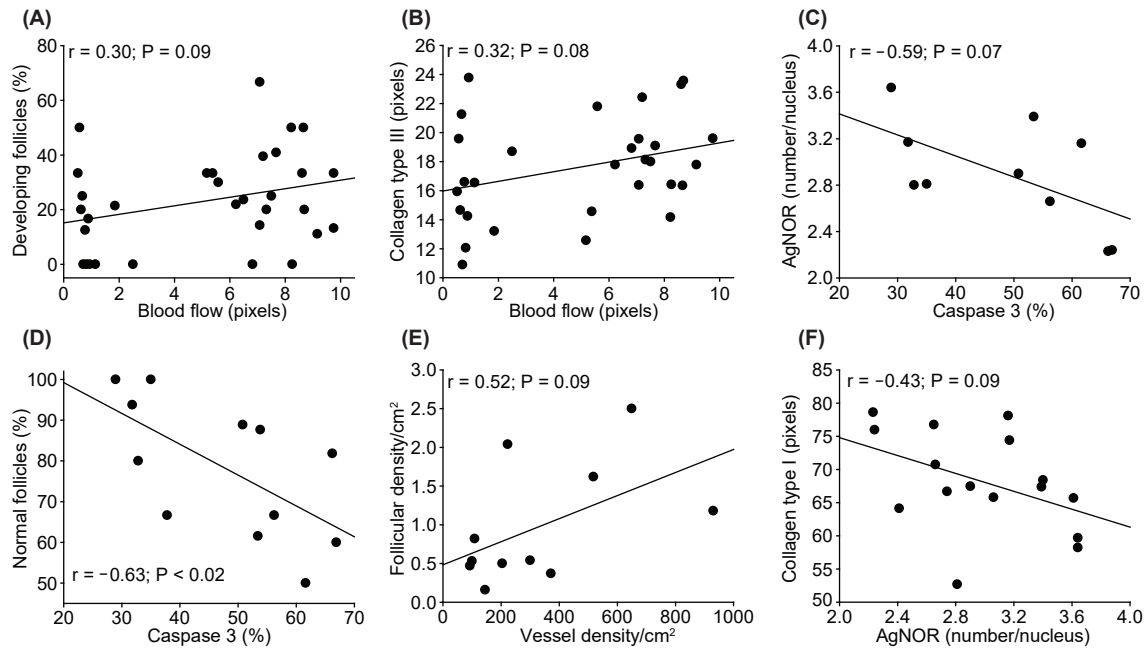

Supplementary Figure 1

Supplement: S1 Fig — (A) Developing follicles and (B) collagen type III fibers versus blood flow area; (C) number of AgNORs and (C) percentage of morphologically normal follicles versus caspase 3; (E) follicular density versus vascular density; and (F) collagen type I fibers versus the number of AgNORs. Each circle on the chart represents an ovarian fragment evaluated. (PDF) [file pone.0241442.s002.pdf]
